# High-Throughput Genomics Enhances Tomato Breeding Efficiency

**DOI:** 10.2174/138920209787581226

**Published:** 2009-03

**Authors:** A Barone, A Di Matteo, D Carputo, L Frusciante

**Affiliations:** Department of Soil, Plant, Environmental and Animal Production Sciences, University of Naples “Federico II”, *Via* Università 100, 80055 Portici, Naples, Italy

**Keywords:** *Solanum lycopersicum*, genetic and genomic resources, molecular markers, microarray, resistance to pathogens, fruit quality.

## Abstract

Tomato (*Solanum lycopersicum*) is considered a model plant species for a group of economically important crops, such as potato, pepper, eggplant, since it exhibits a reduced genomic size (950 Mb), a short generation time, and routine transformation technologies. Moreover, it shares with the other Solanaceous plants the same haploid chromosome number and a high level of conserved genomic organization. Finally, many genomic and genetic resources are actually available for tomato, and the sequencing of its genome is in progress. These features make tomato an ideal species for theoretical studies and practical applications in the genomics field. The present review describes how structural genomics assist the selection of new varieties resistant to pathogens that cause damage to this crop. Many molecular markers highly linked to resistance genes and cloned resistance genes are available and could be used for a high-throughput screening of multiresistant varieties. Moreover, a new genomics-assisted breeding approach for improving fruit quality is presented and discussed. It relies on the identification of genetic mechanisms controlling the trait of interest through functional genomics tools. Following this approach, polymorphisms in major gene sequences responsible for variability in the expression of the trait under study are then exploited for tracking simultaneously favourable allele combinations in breeding programs using high-throughput genomic technologies. This aims at pyramiding in the genetic background of commercial cultivars alleles that increase their performances. In conclusion, tomato breeding strategies supported by advanced technologies are expected to target increased productivity and lower costs of improved genotypes even for complex traits.

## INTRODUCTION

The tomato (*Solanum lycopersicum*) is a worldwide cultivated crop, which is used both as a fresh market and processed product, such as paste, juice, sauce, powder or whole. It is one of the three most important vegetables in the world, with 126 million tons of production in 2007 (FAOSTAT - http://faostat.fao.org/). In the last few years, its global production has increased approximately 10% since for many countries it is a significant source of vitamins and minerals. Furthermore, it has also been recently demonstrated that it is the main source of the carotenoid lycopene, which has antioxidant properties and may help to protect against diseases, such as cancer and cardiovascular disease [1].

Tomato breeding started more than 200 years ago in Europe, with objectives varying according to its use as a fresh or processed product and its cropping system (Table **1**). In particular, in recent years, breeders have focused on addressing consumer complaints regarding tomato flavour, which has been criticised for lack of aroma and taste. Moreover, advances in molecular biology and the development of efficient metabolic engineering tools [2] have prompted research to improve our knowledge of the metabolic pathways involved in synthesising antioxidants and many other health-beneficial compounds in tomato.

Independently of its objectives, tomato breeding has been highly successful during its history since many efforts have been made both by public institutions and private companies to obtain new varieties to meet the needs of farmers, processors and consumers. In the last few years, genomic approaches have increasingly impacted on tomato breeding. In addition, high-throughput genotyping technologies have been able to enhance gene-based selection for better-performing genotypes. This necessarily brief and selective review examines the opportunity that high-throughput genomics offers to bridge the gap between plant physiology and crop sciences. After a short overview of genetics and genomics resources currently available for tomato, it will focus on genomics approaches for improving stress response and increasing fruit quality.

### Genetic Resources

Tomato belongs to the Solanaceae family, which contains about 2800 species and includes many economically important crops, such as potato, pepper and eggplant, as well as ornamental plants such as *Petunia spp.* and *Nicotiana spp.*, and medicinal plants, such as *Datura spp.*, *Capsicum spp.* and *Nicotiana spp*. All tomato species are native to a small area of South America, between Ecuador and Chile, even though they have evolved and are adapted to some of the most diverse and extreme habitats. Traditional tomato genetic resources include nine wild and related species [3], and their collection is publicly available at the Tomato Genetics Resource Center (TGRC, http://tgrc.ucdavis.edu/index.cfm). They are all diploids (2n=2X=24), are similar in chromosome number and structure, and could therefore be used as sources of new genes for tomato breeding. Indeed, given that domestication has narrowed the tomato genetic background, extensive exploitation of variation in germplasm collections is expected according to specific objectives of breeding programs. For some traits, it might be necessary to use wild ancestors of crop tomatoes and to introgress some of the diversity that was lost during domestication to improve yields under optimal as well as stress conditions. Most of the genetic variation present in available wild germplasm has a negative effect on plant adaptation to agricultural constraints; hence, the challenge is to identify and make use only of the advantageous alleles.

In a traditional tomato breeding program based on the use of wild germplasm, *S. lycopersicum* is crossed to wild species to produce F_1_ interspecific hybrids, further used to obtain segregating progenies for molecular and conventional breeding. Through the use of molecular markers, some of these progenies were also used to obtain new genetic resources, such as permanent populations of near-isogenic (NILs) and introgression lines (ILs) [4]. They allow the same genetic stocks to be used worldwide in genetics and genomics applications for tomato breeding. Currently, IL populations exist that derive from wild tomato species such as *S. pennellii, S. habrochaites, S. pimpinellifolium, S. lycopersicoides,* *S. chmielewskii, S. sitiens* [5] and consist of a number of homozygous lines each containing marker defined segments from the wild genome in a uniform cultivated genetic background. These lines have been widely used to localize quantitative trait loci (QTLs) on the molecular map, as shown in Fig. (**1**) and to identify putative genes involved in their genetic control [6]. This has greatly helped the breeding work for these traits, which show a continuous variation and are strongly influenced by environmental conditions.

Additional genetic resources that are particularly interesting to investigate gene function are represented by mutant collections. Among a catalogue list of 1,023 accessions, monogenic mutants, allozyme markers, disease resistance genes and other types of stocks at 625 putative genetic loci are maintained by the Tomato Genetic Resources Center (TGRC) at the University of California - Davis and made available through the TGRC web site (http://tgrc.ucdavis.edu/). In addition, 13,000 M2 families derived from tomato *cv.* M82 through EMS and fast-neutron mutagenesis were visually phenotyped in the field and categorized into a morphological catalog accessible in the Solanaceae Genome Network (SGN) on a site called 'The Genes That Make Tomatoes' (http://zamir.sgn.cornell.edu/mutants/) [7]. The collection consists of 3,417 mutations and includes most of the phenotypes from the monogenic mutant collection of the TGRC. Recently, a γ-irradiated M2 mutant population from the miniature dwarf tomato cultivar “Micro-Tom” has been reported as a contribution to the genetic resources for breeding and functional genomics in tomato [8]. Moreover, an EMS-induced mutation library has been generated from Micro-Tom and mutants are being phenotyped and stored in a pilot database named TOMATOMA (Tomato Mutant Archives) at the National Institute of Genetics (Japan) with the aim of supplying experimental resources and information to research communities [9]. The last two mutant populations come from Micro-Tom, so they are particularly suitable for large-scale and high-throughput mutagenesis due to this genotype small size (approximately 15 cm) and short life cycle (70–90 days) [10]. Because only three major dominant loci (*Dwarf*, *Miniature, *and *Self-Pruning*) are responsible for the miniature growth habit of Micro-Tom, any mutant can be transferred into and tested in the background of any *S. lycopersicum *cultivar by standard crosses.

Finally, very recently the generation of a T-DNA insertional mutagenesis collection was been reported in different tomato cultivars [11]. TILLING mutant populations are also under development in several countries and could be added as useful sources of new genetic stocks. Indeed, tomato TILLING populations have been provided for both cultivated varieties and Micro-Tom [12-14].

### Genomic Resources

Since the first plant genome, that of *Arabidopsis thaliana*, was completely sequenced in 2000, the number of plant genomes that have been sequenced has steadily increased, with some of them also being completed, such as those of rice, poplar and grapevine. The tomato genome is currently being sequenced in the framework of the International Solanaceae Genome Project (SOL) which started at the end of 2004. Detailed information is available in the whitepaper of the SOL consortium (http://www.sgn.cornell.edu/solanaceae-project). *S. lycopersicum*, after Arabidopsis and rice, has one of the smallest plant genomes (900 Mbp). It was chosen as a reference species in the *Solanaceae* since it is also the most intensively investigated genome in this family. Its genome has a high level of synteny with genomes of other species, such as potato, pepper and eggplant, as evidenced through comparative molecular studies. Therefore, the forthcoming tomato genome sequence will provide benefits also to functional genomics of the other *Solanaceae* species, which have been shown to share a well-conserved genomic organization with tomato [15].

The initiative of sequencing the tomato genome has been facilitated by the many genomic resources available for this species. Molecular maps were derived from interspecific crosses between *S. lycopersicum* and *S.* *pennellii*, *S. habrochaites*, *S. pimpinellifollium*, and others are also available [3]. Knowledge of molecular marker positions on tomato maps constitutes the basis for a physical map being constructed by BAC (Bacterial Artificial Chromosomes) clones anchored to the genetic map through molecular markers and by FISHed BAC. In the latter case, many BACs are being physically located on the 12 tomato chromosomes by *in situ* hybridization techniques [16]. Moreover, many EST collections (more than 250,000 EST in all) deriving from various tissues and different developmental stages are also publicly available and integrate information for structural genomics studies [17].

These genomic resources have also been widely exploited for developing new microarray-based technologies. In particular, many arrays are currently supplied worldwide for tomato transcriptome analysis, each combining different technologies and properties, as shown in Table **2**. TOM1 array was developed by the Center for Gene Expression Profiling (CGEP) of the Boyce Thompson Institute for Plant Research (http://bti.cornell.edu/CGEP/CGEP.html) and made publicly available [18, 19]. Each spot on its glass slide corresponds to a single unigene, each selected at random from a number of different cDNA libraries made from a range of tissues including leaf, root, fruit, and flower. To explore the efficiency of technological upgrades in transcriptome analysis, CGEP has recently released a new tomato array called TOM2 array. Moreover, the Affymetrix GeneChip Tomato Genome Array contains 11 probe pairs *per* probe set, spanning the 3’-region of the transcript. The sequence information for this array was selected from public data sources including *Lycopersicon esculentum* UniGene Build #20 (October 3, 2004) and GenBank® mRNAs up to November 5, 2004. TOM1, TOM2 and Affymetrix arrays have already been used for functional genomics studies, whose results are available for the scientific community at the Tomato Functional Genomics Database website (http://ted.bti.cornell.edu). On the other hand, Agilent tomato gene expression microarrays are synthesized on demand as custom microarrays. They harbour features in a higher-density format so that slides can currently be synthesized to cover genome-wide designs, allowing replication of probes on the slide and enhancing accurate estimation of the hybridization signal. Finally, Combimatrix provides TomatArray1.0, including 4x replicates probes designed for Tomato TCs from TIGR *Lycopersicum esculentum* Gene Index Release 11.0 (June 21, 2006). Combimatrix slides can be easily stripped and re-hybridized 4 to 6 times, thereby keeping costs of experiments lower.

Among genomic resources, there are increasing bioinformatics supports for structural and functional genomics for tomato and the *Solanaceae* family in general. SGN (Solanaceae Genome Network, http://www.sgn.cornell.edu) is a website that provides a virtual workbench for researchers working on the *Solanaceae* family, which hosts various sources of data and analysis tools. Other web resources that collect data generated from different tomato “omics” approaches are publicly available and reviewed in Yano and coworkers [20] and Barone and coworkers [17]. However, increasing efforts are required in order to convert raw data into biologically meaningful information.

All these genetics and genomics resources are valuable tools for high-throughput structural and functional genomics applications to achieve important tomato breeding objectives. Such objectives include disease resistance and fruit quality: in-depth knowledge has been attained regarding genes that control these traits and the molecular mechanisms acting in their expression and regulation. In the next sections examples are given of how both structural and functional genomics tools could aid tomato breeding.

## GENOMICS-ASSISTED BREEDING FOR PATHOGEN RESISTANCE

Recent advances in the field of genomics can serve to enhance plant breeding efforts to speed up the release of new varieties. To increase selection efficiency, the use of DNA markers for genetic analysis has become increasingly beneficial, especially when genes under selection are tagged with tightly linked markers. Tomato is very rich in the number and type of molecular markers, most of which are also localized in various molecular maps. Currently, more than 2500 markers, including RFLP, EST, SSR, and COS, are mapped to the twelve tomato chromosomes in a high-density tomato genetic map [21]. Markers and maps available for tomato have been widely used to locate and tag genes or QTLs for disease resistance and many other horticultural characteristics [3]. In particular, disease resistance, often controlled by major genes, has been intensively investigated. Indeed, mapping of resistance genes to many virus, bacterial, nematode and fungal diseases has become an important component of tomato breeding.

To date, more than 40 major resistance genes and many QTLs have been localized on the tomato map, as summarized in Fig. (**2**). Such information is used for more efficient selection through molecular markers (marker-assisted selection, MAS). Many seed companies and public institutions are currently using MAS in tomato breeding for a variety of purposes. These include selection for vertical resistance to viruses, such as tomato/tobacco mosaic virus and tomato spotted virus, to nematodes, and to fungi, such as corky root, *fusarium* and *verticillium* wilt, late blight, and others [3]. MAS is also being used for pyramiding more than one resistance gene [22, 23] in the same variety or breeding line.

Independently of the type of marker developed for each tagged gene, MAS for biotic stress resistance in tomato could be applied successfully in any laboratory without the need for highly sophisticated techniques. The significant progress in automation and robotics may also give the opportunity of greatly increasing selection efficiency in terms of the number of samples contemporaneously analysed for different tagged genes.

More recently, the availability of gene sequence data has led to the development of molecular markers directly from the genes, markers referred to as “genic” molecular markers (GMM) [24]. These markers allow real gene-assisted breeding, without losing the desirable trait due to recombination events between the marker and the gene under selection. In tomato, among the tagged resistant genes (R genes), 16 have so far been cloned and sequenced. Thus, they may represent the subject of more efficient gene-assisted selection, as already reported for the introgression of the resistance genes *Sw-5* [25] and *Ve1* and *Ve2* [26]. The development of new genic markers is today feasible in tomato, as well as in many other crop species, due to the accumulation of EST or gene sequence resources resulting from many large-scale genome or EST sequencing projects initiated in plant species, whose data are being collected in publicly available databases. Indeed, the tomato genome sequencing project is now starting to release the first nucleotide sequences. It is expected that, among these sequences, many new resistance genes will be identified. Polymorphism between resistant and susceptible genotypes will be explored in order to find SNPs or INDELs useful as gene markers. Software tools are also being developed to search for SSR and SNP from EST or gene sequences [27, 28]. Therefore, the increasing availability of random and genic molecular markers so far described for tomato, as well as the enhancement of information on resistance genes deriving from the sequencing of the tomato genome, will facilitate large-scale gene-assisted selection (GAS).

The success of this breeding strategy will also be based on the availability of technological platforms based on large-scale screening through automation. To date, several technologies for automatic large-scale SNP detection have been set up, that have increased levels of resolution in terms of markers analyzed but also the various costs and equipment required, all issues strictly influencing breeder choices [see 29 for a general review]. Since then, other innovative techniques, such as SnaPShot [30], SNPlex [31], SNPWave™ [32], Diversity Arrays Technology (DArT) [33] and pyrosequencing [34], have been developed. Obviously, SNP detection through these automatic systems could be applied to various breeding populations but it first requires considerable work for SNP discovery between resistant and susceptible genotypes, which is performed by re-sequencing target genes in a group of well-phenotyped genotypes.

As for other tomato diseases that are under the genetic control of QTLs, more complex approaches than linkage mapping are required to identify key-genes and markers suitable for assisting selection. The possibility of studying plant transcriptome variations during plant-pathogen interactions through differential approaches, such as cDNA-AFLP, PCR select and others, allowed many ESTs potentially involved in the resistance/tolerance response to be identified in tomato [35, 36]. From these ESTs, some major genes could be fished out and polymorphisms in these genes or their regulation regions could be found between resistant and susceptible genotypes. It will then be possible to design GMMs able to monitor the selection of new resistant varieties.

This approach could be used for all traits that are controlled by many genes with different influence on the phenotype, such as yield, stress tolerance and fruit quality. Specifically, *-*omics technologies could help resolve complex traits in major genes and link higher performing phenotypes to polymorphic QTLs. This may allow phenotype-associated superior allelic combinations in assisted breeding programs to be tracked, although QTLs show low heredity and high interaction with environmental constraints. Examples of -omics applied to plant breeding for quantitative traits aimed at obtaining new improved tomato varieties for fruit quality traits are reported in the next section.

## GENOMICS-ASSISTED BREEDING FOR IMPROVING FRUIT QUALITY

Tomato fruit quality includes several aspects that may be grouped into two categories, namely organoleptic properties and nutritious contents. These are both very complex traits that require the study of various physiological and metabolic components. Indeed, organoleptic quality involves taste and aroma, but also the colour and texture of the fruit and is influenced by varietal differences, the nutritional regime of plants, stage of ripening at harvest and post-harvest storage conditions. Several associations were found between tomato fruit composition and physical characteristics [37] or sensory traits [38], but little is known about their genetic control and the genes responsible for their variation. On the other hand, additional insights are required in the genetic control and environmental regulation of tomato metabolites that cause variations in fruit nutritional quality. This is mainly due to compounds contained in tomato fruits, such as carotenes (mostly lycopene and alpha-carotene, a precursor of vitamin A), ascorbic acid (AsA), and phenolic compounds (flavonoids and hydroxycinnamic acid derivatives).

QTL mapping approaches have been used to localize genomic regions controlling quality traits of processing tomato and a total of 130 QTLs accounting for 38 traits have been mapped so far [3]. These QTLs have been shown to be widely distributed in the genome but mainly concentrated in a few marker-defined chromosome regions. Nevertheless, these regions are still too large and include many other unknown genes that negatively affect the plant phenotype. Therefore, deeper investigations are still required to better define the chromosomal region that control the trait of interest. Furthermore, much research effort has been directed at improving both organoleptic and nutritional quality in tomato fruits through strategies focused mainly on transgenic approaches and on characterizing mutants with pronounced effects on such traits [39]. Consistent with public concerns and policy limitation on widespread cropping of genetically modified plants, alternative approaches to breeding for quality traits are considered desirable.

One such approach could be the tomato genomics-assisted breeding for QTLs described in Fig. (**3**), which combines the use of high-throughput transcriptome analyses with that of new genomics resources, such as the introgression lines. The strategy proposed could help identify candidate genes for QTLs as well as GMMs suitable for a genomics-assisted breeding. For this purpose, the understanding of biological mechanisms controlling complex traits of interest is an essential requirement and the contribution of –omics will allow the identification of major genetic determinants underlying the regulation of the trait expression.

Most studies so far attempted in tomato for dissecting the genetic determinants of fruit quality have focused on ripening processes due to the high magnitude of metabolic changes they entail: ripening confers desirable flavour, colour and texture, increases fruit pathogen susceptibility, imparts numerous quality and nutritional characteristics including fiber content and composition, lipid metabolism and antioxidant composition. The ability to understand key control points in regulating global ripening or within specific ripening processes, such as carotenoid, flavonoid, vitamin biosynthesis, will allow manipulation of nutrition and quality characteristics associated with ripening.

A powerful strategy for identifying genetic regulatory mechanisms in phenotypes of interest is comparative transcriptomics. To elucidate transcriptomic dynamics during expansion, maturation or ripening of the tomato fruit, Alba and coworkers [18] performed a transcriptomics analysis using the microarray TOM1 with nine sequential time-point comparisons both in wild-type and the *Nr*-inhibited ethylene response mutant. This study showed extensive transcript co-regulation in the tomato pericarp. Functional categorization showed that fruit development, hence processes underlying quality determinants, involves many loci encoding translational machinery, transcription factors and signal transduction components, in addition to genes associated with primary metabolism, photosynthesis, cell wall metabolism and hormone responses. Recently, a new approach based on the integration of genomic data sets resulting from diverse technological platforms proved useful for discovering new points of regulatory control of tomato fruit quality-related processes. On combining microarray gene expression analysis and GC-MS metabolomic analysis over small green, breaker and ripe fruit stages interesting correlations involving transcripts and metabolites emerged [40]. These results allowed identification of potential targets for metabolic engineering.

A further example of the transcriptomic approach supporting dissection of complex traits for breeding exploitation in tomato focused on* hp* (high-pigment) mutants that produce higher levels of lycopene and other health-promoting metabolites, such as flavonoids and vitamins [41]. Analysis of global gene expression by microarray approach in the developing fruit of one such mutant [42] allowed candidate genes involved in the regulation of the carotenoid, flavonoid and vitamin (C and E) biosynthetic pathway to be identified.

Consistent with the transcriptomic approach to dissecting genetic contributions to quality-related QTLs, in our laboratory we are currently carrying out experiments to identify transcriptional regulation mechanisms of ascorbate metabolism in fruit. By screening the introgression line (IL) population of *S. pennellii* in the *S. lycopersicon *var*.* M82 genomic background we first confirmed the higher mesocarp ascorbate accumulation of the IL 12-4 over three years and two seasons (spring and summer) in a greenhouse environment. Additionally, IL 12-4 proved significantly lower in fruit firmness and significantly higher in solid soluble content than the M82 reference genotype. Microarray comparative analysis was performed on IL 12-4 and M82 fruit transcriptomes at the red-rip stage by using the Combimatrix TomatArray 1.0 90k. Preliminary results provided evidence of 80 differentially expressed transcripts [43], some of which underlie the key role of the polygalacturonate pathway in providing carbon skeletons for ascorbate biosynthesis. These results are in agreement with those reported for strawberry by Agius and coworkers [44].

Currently, efforts are being focused on the validation by the qPCR approach of transcriptional patterns of the single differentially expressed genes identified by microarray analysis. According to the proposed genomics-assisted breeding approach, complex trait dissection at single candidate gene resolution may be exploited through the detection of polymorphisms associated to the trait. In particular, polymorphic promoters for target genes linked to the fruit ascorbate QTL might be functionally validated for their involvement in the desired phenotype. Once the role of such promoters has been confirmed in fruit ascorbate accumulation, they will be sequenced for marker identification. Associated functional markers may be used for tracking favourable allele combinations in backcrossing progenies aimed at transferring the trait of interest in genomic-assisted breeding programs.

## CONCLUSIONS

Tomato post-genomics holds out the promise of providing comprehensive knowledge of biological processes. Thanks to ongoing genome sequencing, our understanding of the tomato genome has improved, while considerable progress is being made in unravelling gene functions and genome functionality thanks to the growing repertoire of genetic and genomic resources available. Moreover, genomic approaches are expanding the gene pools available for crop improvement and increasing the precision and efficiency with which superior individuals can be identified and selected. Nevertheless, efforts are still required for full exploitation of existing bioinformatics platforms and for developing new ones which enable large scale data sets to be meaningfully managed. In particular, methods for integrating large datasets from different biological systems or provided through different –omics approaches need to be developed. Additionally, the multilayer biology space strictly requires combination with emerging crop modelling to gain predictive power by targeting “breeding by design” [45] in tomato, as well.

In any event, genomics is defining the biotechnological revolution of assisted breeding. We are switching from MAS approaches to breed single marker-defined traits to pyramiding multiple marker-defined traits in the same genotype through MAS. New genomic-assisted approaches are been developed to breed tomato: advances in knowledge on tomato gene structures and functions, combined with new technological tools, will allow conventional plant breeding to be better integrated with genomics, thus leading to the evolution of tomato MAS in genomics-assisted breeding [46, 47].

## Figures and Tables

**Fig. (1):**
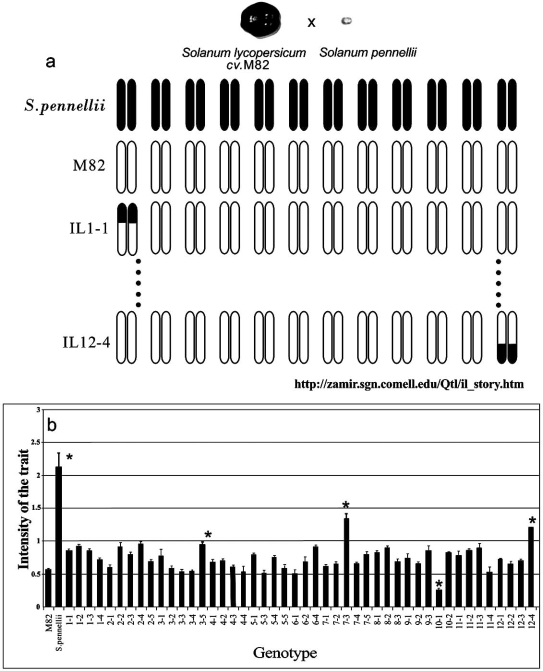
Use of introgression lines (ILs) for QTL identification: a) schematic representation of *S. pennellii* ILs, each carrying a wild homozygous marker-defined chromosomal segment in the genomic background of *S. lycopersicum var.* M82; b) phenotypic profiling of the IL population and their parental lines; asterisks denote three ILs (3-5, 7-3, 12-4) expressing higher levels of the trait of interest, thus evidencing the presence of at least three QTLs which could increase the phenotype under study.

**Fig. (2):**
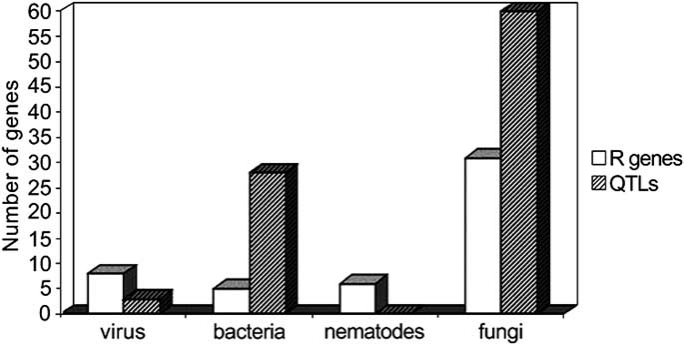
Number of single resistant genes (R) and quantitative trait loci (QTLs) for resistance to pathogens that have been localized on the tomato molecular map.

**Fig. (3):**
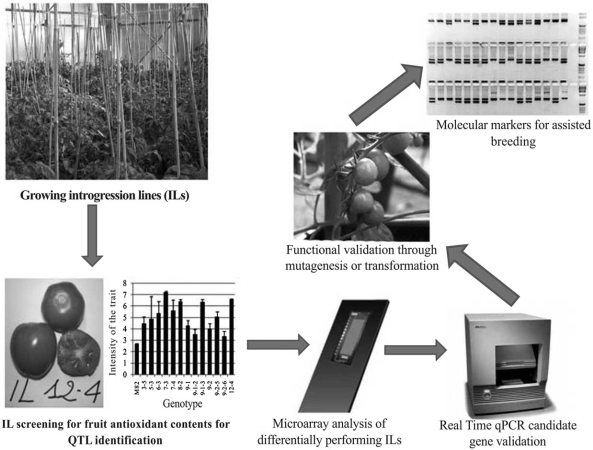
Genomics-based strategy for quantitative trait breeding: studying of comparative transcriptional regulation of fruit antioxidant metabolism in differentially performing introgression lines and design of molecular markers suitable for fruit quality breeding.

**Table 1.** Common and Specific Goals of Tomato Breeding Varying According to Use as Fresh Market or Processing Variety (Modified from Foolad [3])

Universal Goals	Fresh Market CV Goals	Processing CV Goals
Fruit yield	Large, round fruit	Compact, determinate plant habit
Disease resistance	Firmness and shelf-life	Concentrated flowering
Broad adaptability	Uniform fruit size	Fruit set suitable for machine harvest
Earliness in maturity	Fruit shape and colour	Ease of fruit separation from the vine
Ability to set fruit at adverse temperatures	Appearance	Fruit colour
Resistance to rain-induced cracking	Freedom from external blemishes	Fruit pH and total acidity
Tolerance to major ripe-fruit rots	Texture	Total and soluble solids
Adequate vine cover	Taste and flavour	Fruit viscosity

**Table 2:** Available DNA Arrays for Tomato Gene Expression Analysis

Provider	Array	Tomato Probes	Fabrication	Slide
CGEP	TOM1	11,000 cDNA	Spotting cDNAs	glass
CGEP	TOM2	11,000 70-mer oligonucleotides	Printing oligonucleotides	glass
Affymetrix	GeneChip Tomato Genome Array	9,200 25-mer probe sets	Photolithographic *in situ* synthesis	coated quartz wafer
Agilent	Custom Tomato Gene Expression Microarray	Up to 244,000 60-mer oligonucleotides	SurePrint *in situ* synthesis	glass
Combimatrix	TomatArray 1.0	4x 20,200 35-mer oligonucleotides	Phosphoramidite *in situ* synthesis	coated quartz wafer

## ABBREVIATIONS

AFLP: = Amplified Fragment Length Polymorphism
AsA: = Ascorbic Acid
BAC: = Bacterial Artificial Chromosome
COS: = Conserved Ortholog Set
EMS: = Ethylmethane Sulfonate
EST: = Expressed Sequence Tag
FISH: = Fluorescence In Situ Hybridization
GAS: = Gene-Assisted Selection
GC-MS: = Gas Chromatography Mass Spectrometry
GMM: = Genic Molecular Marker
IL: = Introgression Line
INDEL: = Insertion/Deletion
MAS: = Marker-Assisted Selection
NIL: = Near-Isogenic Line
PCR: = Polymerase Chain Reaction
qPCR: = quantitative Polymerase Chain Reaction
QTL: = Quantitative Trait Loci
RFLP: = Restriction Fragment Length Polymorphism
SNP: = Single Nucleotide Polymorphism
SSR: = Simple Sequence Repeat
T-DNA: = Transferred DNA
TILLING: = Targeting Local Lesions IN Genomes
